# Association between axial elongation and corneal power distribution changes induced by aspheric orthokeratology lenses

**DOI:** 10.1186/s40662-025-00453-1

**Published:** 2025-09-28

**Authors:** Mengdi Li, Kailang Zhang, Hua Bi, Xingyi Guo, Lihua Li, Bin Zhang, Xiaoyan Yang

**Affiliations:** 1 Tianjin Eye Hospital, Tianjin Key Lab of Ophthalmology and Visual Science, Tianjin Eye Hospital Optometric Center, No 4. Gansu Rd, Heping District, Tianjin, 300020 China; 2 Nankai University Optometry & Vision Science Institute, Tianjin, China; 3 College of Optometry, Nova Southeastern University, 3301 College Avenue, Davie, FL 33314 USA

**Keywords:** Orthokeratology, Aspheric design, Axial elongation, Corneal power distribution

## Abstract

**Purpose:**

This study investigated how aspheric lens design changes the corneal power distribution and how such changes are associated with the axial elongation in myopic children who underwent orthokeratology.

**Methods:**

This retrospective study of 116 eyes from children aged 8–13 years were enrolled and fitted with three types of lenses: fully spherical lenses (Alpha, n = 45), those with an aspheric alignment curve (AC) and a spherical base curve (BC) (Dreamlite, n = 37), and lenses with a partly aspheric BC and an aspheric AC (Myok, n = 34). Axial lengths were measured at baseline, 6 and 12 months. Corneal topography maps obtained at baseline and after 1 month of lens wear were analyzed with Fourier decomposition: the F0 (spherical), F1 (asymmetry), F2 (regular astigmatism), and F3 (higher-order irregularity) components were extracted and quantified across ten concentric rings with 0.5 mm width.

**Results:**

The 1-year axial elongation was 0.26 ± 0.21 mm, 0.16 ± 0.19 mm, and 0.10 ± 0.19 mm for the Alpha, Dreamlite, and Myok groups, respectively (*P* < 0.001). In the 1-month maps, F0 and F1 peaked at the mid-periphery, and declined peripherally. Dreamlite exhibited F0 values greater than those of Alpha (mean difference: 0.02–0.46 D) within the central 2 mm (*P* < 0.01) and lower than Myok’s values (mean difference: 0.66–1.05 D) in the peripheral 3 to 4.5 mm (*P* < 0.01). Dreamlite also displayed greater F1 compared to Alpha (mean difference: 0.68–0.78 D) within the 1 to 2 mm rings (*P* < 0.01) but showed no significant difference from Myok. F2 and F3 remained flat and small. Three components, F0, F1, and F3, were negatively associated with axial elongation in these children (*P* < 0.001).

**Conclusion:**

Lenses featuring an aspheric AC resulted in reduced axial elongation and increased spherical power and asymmetry in the central cornea, while lenses with a partly aspherical BC improved spherical power in the mid-periphery. A smaller axial elongation was associated with greater post treatment central cornea asymmetry.

## Background

Myopia is a global public health concern with a rising prevalence [1, 2]. It is estimated to affect half the world’s population by 2050, with high myopia reaching a prevalence of 10% [3]. The escalating burden of myopia has led to the growing adoption of orthokeratology lenses in clinical practice for myopia control [4, 5]. It is a rigid contact lens worn overnight, designed to reshape the cornea to provide clear unaided visual acuity during waking hours, and to slow the progression of myopia [6, 7]. The traditional vision-shaping treatment (VST) orthokeratology lens features a four-curve design, comprising the central base curve (BC), reverse curve (RC), alignment curve (AC) and peripheral curve (PC) [7]. The shallow central BC flattens the central cornea to improve visual acuity. The RC, one or more steeply curved zones, surrounds the BC and steepens the mid-periphery of the cornea to induce myopic defocus, which hypothetically slows down axial elongation. Beyond the RC, the AC and PC are designed to align precisely with the corneal surface [8].

While orthokeratology lenses demonstrate significant efficacy in slowing axial elongation (32%–63% reduction compared to single-vision spectacles or soft contact lenses) [9], about a quarter of children fail to achieve satisfactory outcomes [10]. This may be due to factors related to individual factors including genetics, family history, and insufficient outdoor time. It could also suggest that further improvement in lens design is warranted. Optimizing lens parameters to enhance efficacy remains a topic for further investigation [11–13]. Asphericity has recently been introduced into lenses’ BC, AC, or both [14, 15]. Lenses with an aspheric BC demonstrate greater efficacy than those with a spherical BC, one possible reason is that aspheric base curve may induce greater peripheral corneal power change [14]. Children fitted with the Dreamlite lens featuring an aspheric AC exhibited less axial elongation over 1 year compared to those wearing the CRT lens [15]. In multiple comparison studies (Dreamlite vs. Alpha vs. Lucid vs. Euclid), the Dreamlite lens demonstrated the highest efficacy in controlling axial length (AL) progression [16].

However, the mechanism underlying such improved efficacy is unclear. Existing studies mainly compared the axial elongation among different lens designs without exploring potential mechanisms [8, 16, 17]. So far, only one study has reported adding asphericity to the BC results in a more aspheric treatment zone [14]. It is not clear how asphericity changes the corneal power distribution [14, 18]. In spherical lens studies, several factors have been found associated with shorter axial elongation, including the smaller treatment zone [19], larger decentration of the treatment zone [20, 21], larger corneal asymmetry [22], and more accumulated relative corneal refractive power (RCRP) in the central area [23]. Whether such alterations occur after fitting aspheric lenses remains unknown. The answers to these questions are crucial for grasping axial elongation’s underlying mechanism and enhancing future lens design.

In studies comparing corneal topographical changes after lens wear, the lenses being compared often vary in multiple design factors, such as the width of the reverse zone [15], and the back optic zone diameter (BOZD) [22]. Such a design makes isolating specific corneal topographic changes attributed to a single factor impossible. It is essential to design a study with appropriate controls to isolate the corneal modifications caused by a particular factor and demonstrate the combined effects of multiple factors. Furthermore, a method for quantifying the circular nature of corneal power distribution and the center-to-periphery gradient is necessary.

Fourier analysis offers an ideal solution for this purpose [24, 25]. The modulation of the corneal power along the meridians could be decomposed into specific Fourier components. F0 component is the mean value, which can serve as a proxy for the spherical equivalent (SE). F1 is a sinewave running one cycle over 360 degrees, which is closely related to the asymmetry caused by treatment zone decentration. F2 is a sinewave running two cycles over 360 degrees, which represents the magnitude of corneal regular astigmatism [26, 27]. F3 is a sinewave running three cycles over 360 degrees, which represents higher-order irregularity. All three are potentially related to the magnitude of axial elongation. Thus, this study utilized Fourier analysis to quantify the changes in corneal power among children wearing lenses with three different designs. Pairwise comparisons between lenses differing only one factor enable the isolation of impacts of adding asphericity to the base or alignment curves. Lenses with both aspherical base and alignment curves were compared to lenses with spherical designs to emphasize the combined effect. The relationship between isolated or combined corneal changes and axial elongation was also examined. The findings may offer insights into lens design related to corneal profiles and aid in developing future generations of orthokeratology lenses.

## Methods

### Study and participants

All subjects were prescribed VST orthokeratology lenses for myopia control at the Tianjin Eye Hospital Optometric Center from January 2021 to June 2023. The following criteria were set for inclusion into the retrospective analysis: children aged 8 to 13 years with a first-time prescription of orthokeratology lenses; SE in both eyes between − 1.00 and − 5.00 D; astigmatism less than or equal to 2.00 D; spherical anisometropia of 1.50 D or less; and best-corrected visual acuity no worse than 0.0 logMAR for both eyes. The exclusion criteria included a history of ocular diseases or surgical treatments (e.g., constant strabismus, amblyopia, congenital genetic disorders of the eye); prior use of myopia-prevention methods (e.g., low-dose atropine or specially designed spectacles); concurrent use of other treatments during the follow-up period (e.g., low-dose atropine eye drops). All participants were also required to have completed a 1-year follow-up as scheduled. Only data from the right eye was used for analysis. As the data was extracted from de-identified electronic medical records, tthe Institutional Ethical Review Board of Tianjin Eye Hospital (KY2023031) granted a waiver for patient informed consent.

### Lens design

**Fig. 1:**
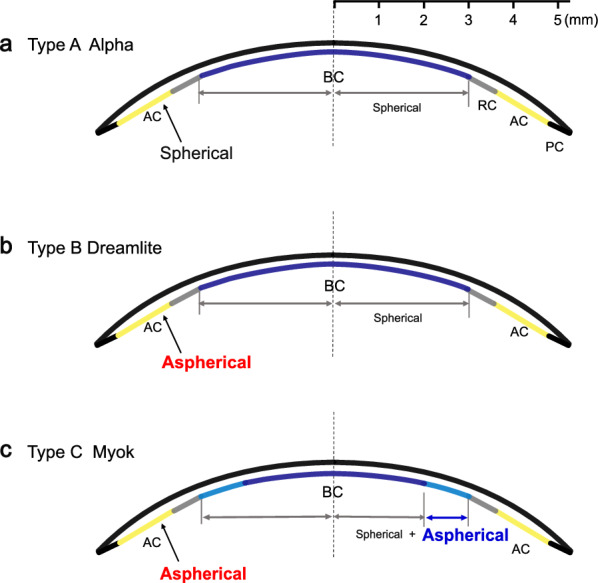
Schematic diagram of the three orthokeratology lens designs. **a** Alpha Lens. **b** Dreamlite Lens. **c** Myok Lens. Blue: base curve (BC); Gray: reverse curve (RC); Yellow: alignment curve (AC); Black: peripheral curve (PC)

All the orthokeratology lenses used in this study had a 4-zone reverse geometry and non-toric design, with BOZD of 6.0 mm, followed by the reverse curve (RC), alignment curve (AC), and peripheral curve (PC). The lens diameter was 10.6 mm. The specifications of the three orthokeratology lenses included in this study are detailed in Table 1 and Fig. 1.

**Table 1:** Characteristics and default parameters of the three orthokeratology lenses

Lens	Material	Dk (10^−11^ cm^2^ × mL O_2_)/(s × mL × mmHg)	BOZD (mm)	RCW (mm)	ACW (mm)	PCW (mm)
Alpha	Boston EM	104	6.0	0.6	1.3	0.4
Dreamlite	Boston XO	100	6.0	0.6	1.25	0.4
Myok	Hexafocon-B	141	6.0	0.6	1.3	0.4

*Dk* = oxygen permeability; *BOZD* = back optic zone diameter; *RCW* = reverse curve width; *ACW* = alignment curve width; *PCW* = peripheral curve width

Type A: Alpha lenses (Alpha Corporation, Japan) utilize a 4-zone reverse geometry design with a spherical profile for each curve. Type B: Dreamlite lenses (Procornea Ltd, The Netherlands) feature a spherical BOZD and an aspherical design in the AC. Type C: Myok lenses (Brighten Optix Ltd, Taiwan) feature a BOZD composed of a 2 mm spherical design combined with a 1 mm aspherical design. Additionally, the AC incorporates an aspherical profile. Professional optometrists fit all lenses with patients, demonstrating good compliance and proper lens care abilities. The children were required to wear the lenses overnight for at least eight hours and come to follow-up examinations on 1 day, 1 week, 1 month, and every 3 months thereafter. All follow-up examinations were conducted between 9:00 a.m. and 12:00 p.m.

### Refraction and AL measurement

Cycloplegic subjective refraction was performed in each group. Cycloplegia was induced 30 min before refraction measurements with 0.1% cyclopentolate hydrochloride eye drops (SINQI, Co., LTD, China). The SE equals the spherical power plus 1/2 cylinder power. AL was measured five times using partial coherence interferometry (AL-Scan, NIDEK, Japan), and the data were automatically averaged. If the minimum difference between two measurements exceeded 0.04 mm, the measurement was repeated. AL was measured at baseline, the 6- and 12-month visit. All patients had no more than 30 days of lens cessation during the study period. All the examination was performed by an experienced optometrist between 9:00 a.m. and 12:00 a.m.

### Tomography parameters and Fourier analysis

All subjects underwent a standard anterior eye and refractive status assessment before commencing orthokeratology lens wear. Corneal topography (TMS-4, Tomey, Nagoya, Japan) was assessed at baseline and every follow-up visit. Children were instructed to fixate on the central light to ensure precise alignment along the visual axis. They were also asked to blink between examinations to maintain tear film integrity. On each measurement occasion, at least four topographic maps were captured, and the map with the best image quality was selected for analysis. Corneal topographic maps with a vertical diameter greater than 8 mm (coverage > 85%) were selected for analysis. The corneal topographer generated the flat corneal curvature (Kf) and steep corneal curvature (Ks) over an 8 mm chord length. The complex corneal power (Fi) change along the 360-meridians could be decomposed into Fourier components for more straightforward interpretation [24, 26, 27].

$${\text{Corneal }}\;{\text{Power}} = {\text{F}}0 + {\text{F1}} \times {\text{sin}}\left( {\alpha - {\text{Phase1}}} \right) + {\text{F2}} \times {\text{sin}}\left( {{2}\alpha - {\text{Phase2}}} \right) + {\text{F3}} \times {\text{sin}}\left( {{3}\alpha - {\text{Phase3}}} \right)$$

where, α is the meridian angle; F0 is the spherical power averaged across the 360-degree meridians. F1 is the asymmetry (tilt or decentration). A larger F1 value indicates that the cornea is more asymmetrical between the two halves (e.g., nasal vs. temporal, top vs. bottom). It is important to note that the magnitude of the F1 value itself can only reveal the magnitude of asymmetry and not its location: the associated phase is necessary to denote where the asymmetry exists. F2 is the regular astigmatism. F3 is a higher-order irregularity component.

**Fig. 2:**
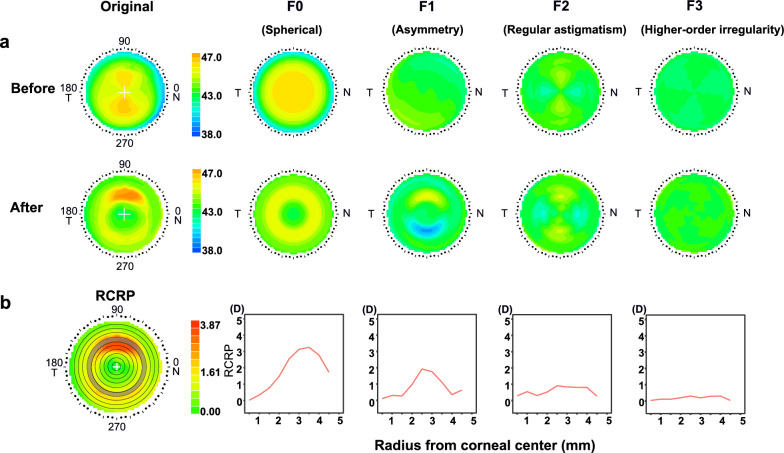
Illustration of Fourier decomposition. **a** An example of corneal topography before and after orthokeratology lens treatment. The original map was decomposed into spherical (F0), asymmetry (F1), regular astigmatism (F2), and higher-order irregularity (F3). **b** The relative corneal refractive power (RCRP) was divided into 10 sections at 0.5 mm intervals. The line chart illustrates the center-to-periphery distribution of mean Fourier parameters across the cornea at these steps

Figure 2a presents the original corneal power maps, before and after lens wearing, alongside Fourier decomposition maps of key components (F0–F3). To highlight the steepening of the peripheral cornea after lens wearing, the RCRP was calculated by subtracting the central corneal refractive power from values in the post-lens-wear map (Fig. 2b). The RCRP map was divided into 10 rings with 0.5 mm intervals further to illustrate the central to periphery gradient of power change. Rings 1 to 4 (0–2 mm radius from the corneal center) were defined as the central corneal zone, while rings 5 to 7 were classified as the mid-peripheral zone. The remaining rings (8 to 10) were designated peripheral corneal zones.

### Treatment zone size and decentration

The treatment zone size and decentration were automatically calculated from the tangential power maps using R software (version 4.4.1, 2024; https://www.R-project.org/). A difference map was generated by subtracting the baseline topography from the topography obtained at the 1-month follow-up. The central region exhibiting a power reduction of more than 0.25 D was identified, and the boundary points were fitted to an ellipse using a custom program developed in the R language, as detailed in previous studies [15, 28]. The center of the fitted ellipse was defined as the center of the treatment zone, and the distance between this center and the corneal vertex was calculated to determine treatment zone decentration. The corneal vertex is the point located at the intersection of the patient’s line of sight and the corneal surface. This is represented by the central corneal light reflex when the cornea is illuminated coaxially with fixation. Additionally, the major and minor axes, along with the orientation of the ellipse, were derived. The treatment zone size was computed as π × major axis × minor axis, expressed in mm^2^.

### Sample size

Based on existing literature [5], a minimum detectable difference of 0.10 mm in AL between the two groups over 1 year was estimated, with a standard deviation (SD) of 0.10 mm. A sample size of 17 subjects per group was required to achieve 80% statistical power (1 − β) at a significance level (α) of 0.05.

### Statistical analysis

For descriptive purposes, the mean value and SD were calculated for all parameters after checking their normality with the Shapiro–Wilk test. A Chi-squared test was used to compare gender among groups. The ANOVA test was used to compare baseline information, including age, SE, SD, CD, Kf, Ks, and AL among groups. The 6-month axial elongation and 1-year axial elongation, treatment zone size and decentration were also evaluated using one-way ANOVA and Tukey HSD post hoc test. The t-test was used to compare the difference between the Fourier parameters between the two groups. Multiple linear regression was used to measure the correlations between the axial elongation and the other variables. Statistical analyses were performed using R Studio (version 4.4.1, https://www.R-project.org/). The level of statistical significance was set at 0.05.

## Results

### Baseline information

A total of 116 subjects were enrolled for analysis. The mean age was 10.26 ± 1.85 years old, with 63 boys and 53 girls. For the refractive features, the mean SE was − 2.70 ± 0.97 D and the mean Ks 43.17 ± 1.34 D. The average AL was 24.71 ± 0.76 mm for all children. There was no significant difference in baseline characteristics amongst all three lens groups (all *P* > 0.05). Detailed information is shown in Table 2.

**Table 2:** Baseline characteristics of each lens group

Parameter	Alpha (n = 45)	Dreamlite (n = 37)	Myok (n = 34)	F/χ^2^	*P*
Age (years)	10.11 ± 2.00	10.48 ± 1.81	10.24 ± 1.71	0.398	0.673*
Sex (M/F)	26/19	18/19	19/15	0.730	0.694^&^
SE (D)	− 2.52 ± 0.85	− 2.79 ± 1.09	− 2.84 ± 0.97	1.325	0.270*
SD (D)	− 2.35 ± 0.78	− 2.63 ± 1.10	− 2.62 ± 0.94	1.164	0.316*
CD (D)	− 0.33 ± 0.36	− 0.32 ± 0.40	− 0.48 ± 0.35	1.591	0.208*
Kf (D)	42.49 ± 1.30	42.57 ± 1.41	42.81 ± 1.14	0.598	0.552*
Ks (D)	43.49 ± 1.52	43.81 ± 1.49	43.94 ± 1.20	1.072	0.346*
AL (mm)	24.75 ± 0.67	24.74 ± 0.94	24.64 ± 0.68	0.228	0.797*

*SE* = spherical equivalent; *SD* = spherical diopter; *CD* = cylinder diopter; *AL* = axial length; *Kf* = flat curvature of the cornea; *Ks* = steep curvature of cornea; *D* = diopter

^&^indicates Chi-squared test, *indicates one-way ANOVA

### Comparison of axial elongation for three orthokeratology lens design groups

**Fig. 3:**
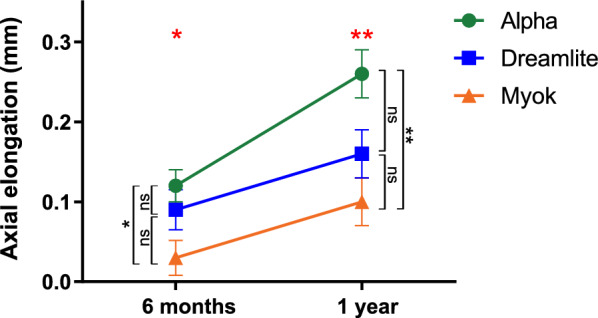
Comparison of axial elongation according to the three orthokeratology lens groups for 6 months and 1 year. Green: Alpha lens; Blue: Dreamlite lens; Orange: Myok lens. Red stars indicate significant differences between all groups: ns, not significant, * *P* < 0.05, ** *P* < 0.01; Black stars indicate significant differences between two groups: ns, not significant, * *P* < 0.05 and ** *P* < 0.01

After 6 months of treatment, axial elongation was 0.12 ± 0.14 mm in the Alpha group, 0.09 ± 0.15 mm in the Dreamlite group, and 0.03 ± 0.13 mm in the Myok group. Statistically significant differences were identified among the three groups (*P* = 0.028, Fig. 3). In pairwise comparison, a significant difference was observed only between the Myok and Alpha groups, representing the combined effect of aspheric AC + BC (*P* = 0.031). Similar results were found at the 1-year follow-up. The 1-year axial elongation was found to be 0.26 ± 0.21 mm, 0.16 ± 0.19 mm, and 0.10 ± 0.19 mm in the Alpha, Dreamlite, and Myok groups, respectively (*P* < 0.001). Again, there was a significant difference in the 1-year axial elongation between the Myok and Alpha groups (*P* = 0.004), demonstrating that asphericity in both the AC and BC produced a robust interaction effect.

### The Fourier parameter values among the three lens groups

**Fig. 4:**
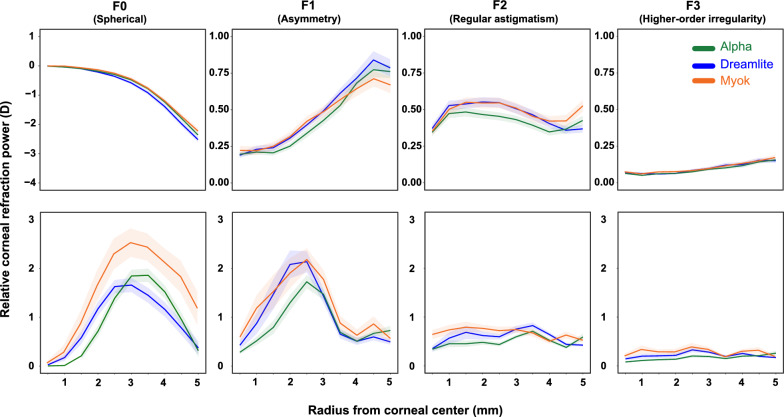
Corneal Fourier parameter distributions across three orthokeratology lens groups. The top panels display baseline data, while the bottom panels show data from the 1-month follow-up. Green: Alpha lens; Blue: Dreamlite lens; Orange: Myok lens. Shaded region indicates 95% confidence interval

The RCRP for three different types of lenses is summarized in Fig. 4. At baseline, the Fourier components extracted from the RCRP were similar across the three lenses. From center to periphery, the spherical power (F0) decreased from 0 to approximately − 2.45 D at 5 mm, illustrating the typical steep center and flatter periphery pattern. Although asymmetry (F1) increased from center to periphery, the overall value remained small (less than 1 D) in all groups. Regular astigmatism (F2) and higher-order aberration (F3) remained consistent from center to periphery.

After treatment, central corneal flattening and mid-peripheral steepening were evident in all three lens types. Spherical power (F0) values peaked at the mid-periphery (2.5–3.5 mm) before decreasing toward the periphery. Such relative positive power in the mid-periphery, typically about 1.5–2.5 D, is intended to induce myopic defocus on the retina and potentially slow axial elongation. Corneal asymmetry (F1) showed a similar pattern, peaking at 2–2.5 mm. Notably, the maximum corneal asymmetry was about 2 D, more than double the baseline value. After lens wear, regular astigmatism (F2) and higher-order aberrations (F3) remained small and stable. Detailed numbers are presented in Table 3.

**Table 3:** The Fourier parameter values among three lens groups at baseline and one-month visit

	F0 (Spherical, D)			F1 (Asymmetry, D)			F2 (Regular astigmatism, D)			F3 (Higher-order irregular, D)		
	Alpha	Dreamlite	Myok	Alpha	Dreamlite	Myok	Alpha	Dreamlite	Myok	Alpha	Dreamlite	Myok
Baseline (ring)												
1	− 0.01 ± 0.02	0.00 ± 0.02	− 0.01 ± 0.03	0.20 ± 0.10	0.19 ± 0.11	0.22 ± 0.18	0.35 ± 0.16	0.37 ± 0.16	0.35 ± 0.18	0.06 ± 0.03	0.07 ± 0.04	0.07 ± 0.05
2	− 0.04 ± 0.12	− 0.02 ± 0.13	− 0.01 ± 0.15	0.21 ± 0.11	0.23 ± 0.15	0.22 ± 0.18	0.47 ± 0.22	0.53 ± 0.20	0.50 ± 0.21	0.05 ± 0.03	0.06 ± 0.07	0.06 ± 0.04
3	− 0.09 ± 0.20	− 0.09 ± 0.18	− 0.07 ± 0.19	0.21 ± 0.13	0.24 ± 0.15	0.25 ± 0.17	0.48 ± 0.21	0.54 ± 0.19	0.55 ± 0.19	0.06 ± 0.03	0.06 ± 0.04	0.07 ± 0.05
4	− 0.18 ± 0.24	− 0.21 ± 0.20	− 0.14 ± 0.24	0.25 ± 0.13	0.31 ± 0.14	0.32 ± 0.19	0.47 ± 0.19	0.55 ± 0.20	0.54 ± 0.21	0.06 ± 0.03	0.06 ± 0.04	0.07 ± 0.05
5	− 0.30 ± 0.29	− 0.36 ± 0.25	− 0.26 ± 0.28	0.34 ± 0.19	0.40 ± 0.14	0.42 ± 0.20	0.45 ± 0.18	0.55 ± 0.20	0.54 ± 0.21	0.07 ± 0.03	0.08 ± 0.05	0.08 ± 0.05
6	− 0.48 ± 0.34	− 0.58 ± 0.32	− 0.45 ± 0.35	0.43 ± 0.21	0.49 ± 0.15	0.49 ± 0.23	0.43 ± 0.18	0.51 ± 0.20	0.51 ± 0.21	0.09 ± 0.06	0.10 ± 0.07	0.09 ± 0.06
7	− 0.78 ± 0.41	− 0.92 ± 0.41	− 0.76 ± 0.37	0.53 ± 0.24	0.61 ± 0.20	0.57 ± 0.28	0.39 ± 0.18	0.47 ± 0.21	0.45 ± 0.21	0.10 ± 0.06	0.12 ± 0.10	0.11 ± 0.08
8	− 1.23 ± 0.50	− 1.38 ± 0.49	− 1.19 ± 0.41	0.68 ± 0.30	0.72 ± 0.29	0.65 ± 0.30	0.35 ± 0.17	0.41 ± 0.18	0.42 ± 0.19	0.12 ± 0.09	0.13 ± 0.10	0.13 ± 0.07
9	− 1.78 ± 0.53	− 1.97 ± 0.53	− 1.72 ± 0.53	0.77 ± 0.34	0.84 ± 0.37	0.71 ± 0.30	0.37 ± 0.19	0.36 ± 0.15	0.42 ± 0.23	0.14 ± 0.08	0.15 ± 0.10	0.15 ± 0.09
10	− 2.36 ± 0.53	− 2.52 ± 0.53	− 2.23 ± 0.57	0.76 ± 0.30	0.79 ± 0.36	0.67 ± 0.34	0.43 ± 0.22	0.37 ± 0.15	0.52 ± 0.23	0.16 ± 0.07	0.15 ± 0.10	0.17 ± 0.10
One month (ring)												
1	0.00 ± 0.03	0.03 ± 0.06	0.06 ± 0.17	0.28 ± 0.25	0.43 ± 0.56	0.59 ± 0.68	0.34 ± 0.33	0.35 ± 0.38	0.63 ± 0.54	0.08 ± 0.06	0.14 ± 0.19	0.19 ± 0.25
2	0.01 ± 0.20	0.17 ± 0.40	0.27 ± 0.82	0.52 ± 0.56	0.87 ± 1.32	1.18 ± 1.55	0.45 ± 0.47	0.57 ± 0.73	0.73 ± 0.59	0.11 ± 0.12	0.20 ± 0.31	0.33 ± 0.48
3	0.21 ± 0.53	0.58 ± 0.80	0.86 ± 1.51	0.79 ± 0.91	1.47 ± 1.87	1.52 ± 2.01	0.45 ± 0.45	0.69 ± 0.84	0.78 ± 0.66	0.13 ± 0.15	0.20 ± 0.27	0.28 ± 0.30
4	0.72 ± 0.78	1.18 ± 0.87	1.64 ± 1.84	1.30 ± 1.06	2.08 ± 1.74	1.90 ± 1.80	0.48 ± 0.33	0.62 ± 0.51	0.76 ± 0.43	0.14 ± 0.13	0.22 ± 0.28	0.28 ± 0.37
5	1.39 ± 0.84	1.63 ± 0.83	2.29 ± 1.85	1.73 ± 0.91	2.14 ± 1.32	2.18 ± 1.32	0.44 ± 0.25	0.60 ± 0.34	0.71 ± 0.47	0.20 ± 0.23	0.33 ± 0.29	0.38 ± 0.47
6	1.85 ± 0.86	1.66 ± 0.88	2.52 ± 1.70	1.47 ± 0.75	1.43 ± 0.87	1.77 ± 1.12	0.59 ± 0.26	0.75 ± 0.41	0.73 ± 0.40	0.19 ± 0.19	0.28 ± 0.18	0.33 ± 0.33
7	1.86 ± 0.92	1.45 ± 0.94	2.43 ± 1.62	0.69 ± 0.51	0.65 ± 0.56	0.87 ± 0.87	0.71 ± 0.33	0.83 ± 0.38	0.67 ± 0.44	0.15 ± 0.12	0.19 ± 0.16	0.18 ± 0.15
8	1.52 ± 0.90	1.16 ± 0.90	2.13 ± 1.64	0.51 ± 0.35	0.51 ± 0.46	0.62 ± 0.70	0.52 ± 0.26	0.64 ± 0.26	0.49 ± 0.30	0.20 ± 0.14	0.25 ± 0.18	0.29 ± 0.25
9	0.95 ± 0.95	0.78 ± 0.87	1.82 ± 1.92	0.67 ± 0.35	0.60 ± 0.44	0.85 ± 0.92	0.38 ± 0.20	0.44 ± 0.20	0.62 ± 0.41	0.21 ± 0.16	0.19 ± 0.13	0.31 ± 0.39
10	0.31 ± 0.88	0.37 ± 0.83	1.18 ± 1.98	0.73 ± 0.64	0.50 ± 0.34	0.56 ± 0.60	0.59 ± 0.49	0.42 ± 0.16	0.52 ± 0.28	0.26 ± 0.34	0.17 ± 0.09	0.18 ± 0.15

*SD* = standard deviation; *D* = diopter

Although the overall RCRP shapes after treatment were similar, clear quantitative differences were observed among the three lens types. To highlight the effects attributable specifically to asphericity applied to the BC or AC, we conducted a series of pairwise comparisons.

### Comparison of Fourier parameters between the Dreamlite and Alpha groups

**Fig. 5:**
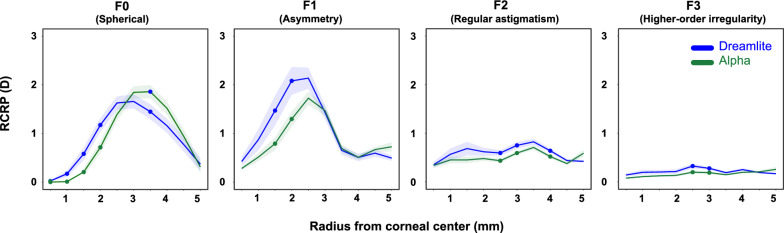
Effect of an aspheric alignment curve: comparison of corneal Fourier parameters between the Dreamlite and Alpha groups at the 1-month visit. Green: Alpha lens; Blue: Dreamlite lens. The solid points indicate statistically significant differences between the two groups. Shaded region indicates the 95% confidence interval. RCRP, relative corneal refraction power; D, diopter

After 1 month of lens wear, RCRP differences between the Dreamlite and Alpha groups were mainly located in the central cornea (Fig. 5). With F0 (spherical power) values in the Dreamlite group peaking at 3 mm (1.66 ± 0.88 D) and those in the Alpha group peaking at 3.5 mm (1.86 ± 0.92 D), F0 (spherical power) was significantly greater in the Dreamlite group within the central 2 mm (*P* < 0.05). The Alpha group’s value rose higher, around 3.5 mm (*P* < 0.05). In the far periphery, values in both groups are similar. This shows that Dreamlite’s aspheric AC produced a faster-rising slope of myopic defocus in the central cornea. F1 (asymmetry) values in both groups peaked at 2.5 mm, and the values were significantly greater in the Dreamlite group in the rings from 1 to 2 mm (*P* < 0.05). The amount of regular astigmatism (F2) and higher-order aberrations (F3) remained small and flat. There were only significant differences at sporadic locations in the mid-periphery (*P* < 0.05). Detailed statistics are presented in Table 3.

### Comparison of Fourier parameters between the Dreamlite and Myok groups

**Fig. 6:**
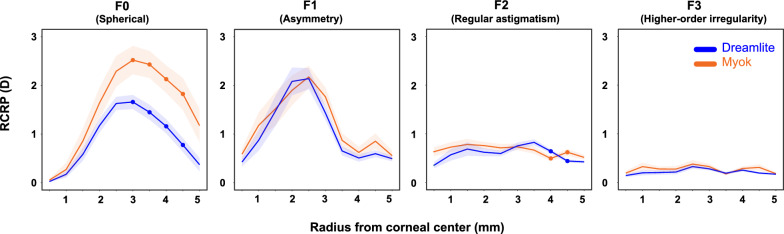
Effect of an aspheric base curve: comparison of corneal Fourier parameters between the Dreamlite and Myok groups at the 1-month visit. Orange: Myok lens; Blue: Dreamlite lens. The solid points indicate statistically significant differences between the two groups. Shaded region indicates the 95% confidence interval. RCRP, relative corneal refraction power; D, diopter

Myok and Dreamlite RCRP profiles differed only in the mid-periphery and periphery (Fig. 6). Both groups' F0 (spherical power) peaked around 3.0 mm (2.52 ± 1.70 D for Myok, 1.66 ± 0.88 D for Dreamlite). F0 (spherical power) values were significantly greater in the Myok group in the rings beyond 3 mm (*P* < 0.05), and the difference within the central 2.5 mm was insignificant. This shows that an aspheric BC changes the magnitude of myopic defocus to the peripheral cornea. Both groups’ F1 (asymmetry) values peaked at 2.5 mm before declining towards the periphery. There were no significant differences between the two groups across the cornea. Regular astigmatism (F2) and higher-order aberration (F3) remained small and flat. Myok and Dreamlite groups showed similar values across the entire cornea, with occasional significant differences in mid-periphery in F2 (*P* < 0.05). Detailed statistics are presented in Table 3.

### Comparison of Fourier parameters between the Myok and Alpha groups

**Fig. 7:**
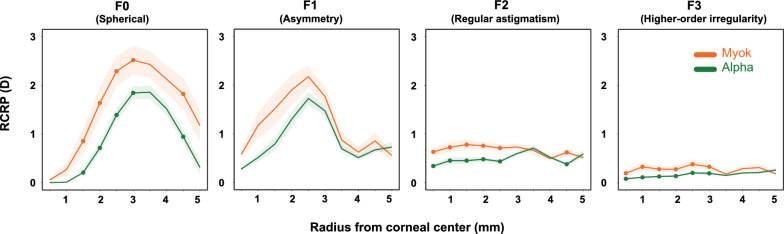
Combined effect of aspheric alignment and base curves: comparison of corneal Fourier parameters between the Myok and Alpha groups at the 1-month visit. Orange: Myok lens; Green: Alpha lens. The solid points indicate statistically significant differences between the two groups. Shaded region indicates the 95% confidence interval. RCRP, relative corneal refraction power; D, diopter

The difference in RCRP between Myok and Alpha was found in both the central and the periphery, mirroring what would have been predicted by summing the individual effects of AC and BC. Figure 7 shows that both groups' F0 (spherical power) peaked around 3.0 mm (2.52 ± 1.70 D for Myok, 1.86 ± 0.92 D for Alpha); however, the F0 (spherical power) values were significantly larger in the Myok group in both the central (1.5–3 mm rings, *P* < 0.05), and the peripheral cornea (rings around 4.5 mm, *P* < 0.05). Both groups' F1 (asymmetry) values peaked at 2.5 mm before declining towards the periphery. Although F1 values in the Myok group appeared greater, the differences were insignificant. Myok group demonstrated significantly greater regular astigmatism (F2) in the central 2.5 mm and 4.5 mm ring at the periphery (*P* < 0.05). The Myok group also showed significantly greater high-order aberration (F3) in the central 3 mm (*P* < 0.05).

### Treatment zone comparison

**Fig. 8:**
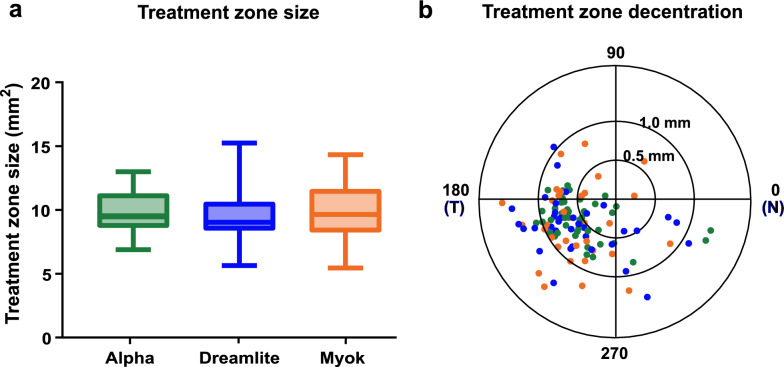
Treatment zone parameters. **a** Treatment zone size. **b** Treatment zone decentration in a polar plot. Green: Alpha lens; Blue: Dreamlite lens; Orange: Myok lens

There was no significant difference in treatment zone size between the Alpha (9.84 ± 1.48 mm^2^), Dreamlite (9.51 ± 1.99 mm^2^), and Myok (9.79 ± 2.14 mm^2^, *P* = 0.697) lenses (Fig. 5a). The decentration magnitude was slightly larger in Dreamlite (0.84 ± 0.28 mm) and Myok (0.83 ± 0.31 mm) lenses compared to the eyes with Alpha lens treatment (0.69 ± 0.25 mm, *P* = 0.023). A significant difference was observed only between the Dreamlite and Alpha groups in pairwise comparisons (*P* = 0.040). In all three groups, the treatment zone is mainly decentered on the temporal inferior quadrant. There was also no difference in decentration direction (213.12 ± 48.43 degrees for Alpha vs. 224.14 ± 48.43 degrees for Dreamlite vs. 198.44 ± 60.49 degrees for Myok, *P* = 0.098) (Fig. 8).

### Multiple linear regression for axial elongation and Fourier parameters

Our final analysis explored the association between Fourier parameters across 10 rings and the 1-year axial elongation. The other factor that affects axial elongation in myopic children is age. Therefore, a multiple linear regression was performed between axial elongation and each Fourier component, after controlling for baseline age. Table 4 summarizes the results from such analyses. First, Fourier parameters tended to be significantly associated with axial elongation within the central area (rings 2 to 4, 0.5–2 mm). Beyond ring 5 (2–2.5 mm), significant association only sporadically appeared. Second, among the four Fourier parameters, F0, F1, and F3 appeared to be consistently associated with axial elongation. Last, negative beta values indicated that larger Fourier values were associated with smaller axial elongations.

**Table 4:** The relationship between the Fourier parameters and 1-year axial elongation

Rings	F0 (β/*P*)	F1 (β/*P*)	F2 (β/*P*)	F3 (β/*P*)
1	− 0.275	− 0.085	− 0.064	− 0.279
	0.126	0.015*	0.127	0.005**
2	− 0.076	− 0.034	− 0.042	− 0.132
	0.026*	0.024**	0.181	0.016*
3	− 0.054	− 0.032	− 0.070	− 0.189
	0.002**	0.003	0.010**	0.010*
4	− 0.045	− 0.040	− 0.064	− 0.152
	0.001**	0.002**	0.130	0.023*
5	− 0.031	− 0.025	− 0.005	− 0.125
	0.031*	0.109	0.924	0.019*
6	− 0.014	− 0.003	− 0.119	− 0.107
	0.363	0.878	0.018*	0.159
7	− 0.006	− 0.015	− 0.082	0.063
	0.678	0.589	0.085	0.634
8	− 0.009	− 0.069	− 0.083	− 0.191
	0.569	0.052	0.214	0.041*
9	− 0.017	− 0.037	− 0.092	− 0.138
	0.246	0.255	0.157	0.076
10	− 0.024	− 0.015	0.010	0.076
	0.137	0.691	0.864	0.382

**indicates *P* < 0.01, * indicates *P* < 0.05. Rings 1 to 4 represent the central corneal zone, rings 5 to 7 correspond to the mid-peripheral zone, and rings 8 to 10 denote the peripheral corneal zone

## Discussion

### Summary

In this study, Fourier decomposition was used to analyze the changes in corneal power induced by asphericity and to determine how these changes were related to axial elongation. Compared with the Alpha group (traditional spherical lens), the Dreamlite group (with an aspherical AC) exhibited greater spherical asymmetry in the mid-periphery (within a 2.5 mm radius from the corneal center). In contrast to the Dreamlite group, the mean RCRP in the mid-peripheral cornea in the Myok group (part of BC with an aspherical curve) increased. Each type of orthokeratology lens proved effective, with Myok, which has both aspherical BC and AC, demonstrating superior myopia control. This suggests that the combination of both a faster-rising slope and greater magnitude of myopic defocus, evidenced by F0 values, may be mechanisms underlying the success of such lenses in slowing myopic progression.

### Axial elongation related to previous studies

Our findings indicated that all three orthokeratology lenses effectively controlled myopia in children. The 1-year axial elongation in the Alpha group was 0.26 ± 0.21 mm, below the commonly accepted threshold for rapid myopia progression (≥ 0.36 mm per year) [29]. The other two groups exhibited significantly greater myopia control, with axial elongation values of 0.16 ± 0.19 mm in the Dreamlite group and 0.10 ± 0.19 mm in the Myok group (*P* < 0.001). Consistent with previous studies, the average 1-year axial elongation was 0.17 ± 0.02 mm for Dreamlite and 0.22 ± 0.02 mm for Alpha [16]. Notably, the Myok lens demonstrated favorable myopia control efficacy. Orthokeratology lenses with an aspherical base curve demonstrated a 1-year axial elongation of 0.19 ± 0.20 mm [18], which was slightly lower than the results of this study. This suggests that the enhanced myopia control effect of the Myok lens may be attributed to its dual aspheric design involving both the BC and the AC. Furthermore, previous studies have defined a 1-year axial elongation of less than 0.1 mm as a successful outcome for orthokeratology lens treatment [30]. The Myok group also showed promising results within this threshold.

### Fourier analysis findings and AC asphericity

Over the central 3 mm area (1.5 mm radius), the corneal asymmetry significantly increased from 0.23 ± 0.07 D before the procedure to 0.73 ± 0.39 D at 12 months afterward (*P* < 0.0001). Higher-order irregularity increased significantly from 0.10 ± 0.02 D to 0.13 ± 0.05 D (*P* < 0.0032). This finding aligns with previous studies that have found that orthokeratology treatment can increase asymmetry and higher-order irregularity [22, 31]. Furthermore, this current study demonstrated that incorporating asphericity into the AC curve can increase the asymmetry of the central and mid-periphery cornea (2.5 mm radius). To better understand the relationship between aspherical AC and post-treatment corneal asymmetry, we analyzed post-treatment RCRP at 0.5 mm intervals. It is known that the AC typically resides within a 4 mm radius of the corneal center. In orthokeratology lenses, the curvatures of the BC and AC are generally interdependent. Therefore, modifying one of these curves may not confine corneal power changes to the intended location but may influence the overall corneal power distribution. The added asphericity makes the AC curve more similar to corneal curvature in the periphery, which changes the entire spatial distribution of hydrostatic pressure. We speculate that such changes in pressure distribution facilitate the accumulation of epithelial cells migrating from the central cornea.

### Fourier analysis findings and BC asphericity

Our study also compared orthokeratology lenses featuring partially aspherical BC (Myok) to those with a spherical BC (Dreamlite). With the added asphericity to BC, the mean spherical component (F0) in RCRP was increased in the central corneal area within a 3 mm radius, although this increment did not reach a significant level. Beyond the 3 mm radius, the difference was further amplified, with the Myok lens inducing a significantly greater F0 component. We speculate that the transition zone between the BC and RC was designed with an aspherical profile, which is hypothesized to enhance the curvature difference between BC and RC. This may generate greater negative pressure, increasing epithelial redistribution and accumulation in the RC zone. This results in a significant increase in mid-peripheral corneal refractive power, as observed in corneal topography. This matched well with a previous study reporting that the lenses with aspheric base curve group exhibited a significantly greater mean change in RCRP in corneal areas beyond the central 3 mm radius [14]. However, within the central 3 mm radius, the temporal and superior sides were significantly greater than those of the lenses with spherical base curve group [14]. One notable point is that the aspherical BC design did not increase central corneal asymmetry (F1).

### Advantage in methods

This study applied Fourier analysis to assess detailed corneal power changes with smaller intervals. Previous studies have typically used 3.0 mm or 6.0 mm central corneal zones to evaluate morphological changes following orthokeratology and their impact on visual quality [31, 32]. However, our findings reveal significant differences in Fourier parameters between these zones after treatment. The 3.0 mm zone primarily represents the treatment zone area, which remains relatively flat, whereas the 6.0 mm zone encompasses the reverse and alignment curves, reflecting broader corneal reshaping. Therefore, a single value may not fully capture the corneal modifications induced by orthokeratology. Similar to orthokeratology-induced corneal reshaping, Haris et al. [33] quantified post-refractive surgery corneal changes using 2 mm intervals and found that FS-LASIK induced greater regular astigmatism, irregularities, and more pronounced mid-peripheral (8 mm) flattening of the anterior and total cornea compared to SMILE. This study utilized a finer 0.5 mm interval, enabling a more precise assessment of corneal morphological changes following orthokeratology lens wear. This approach comprehensively evaluates corneal power distribution and the center-to-periphery gradient.

Additionally, using this method demonstrated that lenses with aspheric designs exhibited larger mean values of Fourier parameters and tended to show greater variability, as reflected by wider standard deviations. Therefore, it is important to monitor individual patients more closely when using such lens designs.

### Study design advantage

The study aims to highlight the effect of aspherical additions to different curves on myopia control with orthokeratology. This was carefully implemented through several design features. First, we intentionally kept all lenses at the same back optical zone diameter of 6.0 mm. The BOZD of lenses is associated with treatment zone size and decentration, two critical factors related to axial elongation [5, 20, 34, 35]. A single BOZD eliminates the confounding interaction between changes in the treatment zone and asphericity. Second, our analysis utilized a paired comparison design, allowing us to isolate the individual BC or AC aspherical designs on corneal refractive power distribution and reveal the combined effect. Therefore, the findings of this study can provide insight into how modifying different curve segments of the VST design enhances efficacy. Third, corneal changes generally stabilize within 1 month of orthokeratology lens wear, as observed in corneal topography [22, 36]. In several previous studies [20, 23], the RCRP summed over the central 4 mm area values was used to represent total corneal changes. This study utilized the 1-month corneal changes to predict 1-year myopia progression, identifying spherical power and asymmetry within a 1 mm or 2 mm central radius as potential early indicators of lens efficacy for clinical application.

### Limitations

This study had several limitations. Firstly, the current 1-year follow-up duration remains relatively limited for evaluating long-term treatment effects. Extended longitudinal observation over the next 2 years is planned to further characterize retinal defocus changes and their association with axial elongation. Secondly, the impact of increased asymmetry and higher-order aberrations on image quality was not evaluated; a future study will focus on assessing visual and optical quality thresholds. Thirdly, factors such as pupil size, accommodative lag, and peripheral refractive profile, which may influence the myopia control efficacy of orthokeratology, were not considered. Lastly, the peripheral retinal refraction distributions induced by the three lens designs were not measured. However, corneal refractive changes were used to predict their correlation with AL changes. We aimed to predict axial growth through short-term corneal changes, providing a basis for optimizing and shortening the orthokeratology lens adjustment period, which will be explored in future prospective studies.

## Conclusion

Orthokeratology treatment significantly reshaped the spatial distribution of corneal power changes in myopic eyes, affecting both the central and mid-peripheral regions. Lenses with an aspherical alignment curve increased spherical power and central corneal asymmetry. In contrast, lenses with a partially aspherical base curve significantly enhanced spherical power in the mid-periphery, potentially improving myopia control through more targeted defocus. Eyes with smaller axial elongation exhibited greater mid-peripheral asymmetry and higher-order irregularities, suggesting a link between slower myopia progression and corneal shape changes. These findings underscore the importance of lens design in optimizing corneal power distribution and provide guidance for future lens modifications to enhance efficacy while maintaining visual quality.

## Data Availability

The data that support the findings of this study are available from the corresponding author upon reasonable request.
